# Elevated C-reactive protein is associated with lower increase in knee muscle strength in patients with knee osteoarthritis: a 2-year follow-up study in the Amsterdam Osteoarthritis (AMS-OA) cohort

**DOI:** 10.1186/ar4580

**Published:** 2014-06-13

**Authors:** Diana C Sanchez-Ramirez, Marike van der Leeden, Martin van der Esch, Leo D Roorda, Sabine Verschueren, Jaap H van Dieën, Joost Dekker, Willem F Lems

**Affiliations:** 1 Amsterdam Rehabilitation Research Center | Reade, PO Box 58271, Amsterdam 1040 HG, The Netherlands; 2 MOVE Research Institute Amsterdam, Faculty of Human Movement Sciences, VU University Amsterdam, De Boelelaan 1105, 1081 HV Amsterdam, The Netherlands; 3 KU Leuven, Department of Rehabilitation Sciences, Tervuursevest 101, 3001 Leuven, Belgium; 4 VU University Medical Center, Department of Rehabilitation Medicine, De Boelelaan 1117, 1081 HZ Amsterdam, The Netherlands; 5 King Abdulaziz University, Abdullah Sulayman, Jeddah 22254, Saudi Arabia; 6 VU University Medical Center, Department of Psychiatry, Amsterdam, De Boelelaan 1117, 1081 HZ Amsterdam, The Netherlands; 7 VU University Medical Center, Department of Rheumatology, Amsterdam, De Boelelaan 1117, 1081 HZ Amsterdam, The Netherlands; 8 Jan van Breemen Research Institute | Reade, PO box: 58271, 1040 HG Amsterdam, The Netherlands

## Abstract

**Introduction:**

The aim of this study was to examine the associations of elevated serum C-reactive protein (CRP) and erythrocyte sedimentation rate (ESR) with change in muscle strength in patients with established knee osteoarthritis (OA), at 2 years.

**Methods:**

Data from 186 patients with knee OA were gathered at baseline and at 2-year follow-up. CRP (in milligrams per liter) and ESR (in millimeters per hour) were measured in serum from patients’ blood. Strength of quadriceps and hamstrings muscles was assessed by using an isokinetic dynamometer. The association of inflammatory markers with change in knee muscle strength was analyzed by using uni- and multi-variate linear regression models.

**Results:**

Patients with elevated CRP values at both baseline and 2-year follow-up exhibited a lower increase in knee muscle strength for a period of 2 years (β = -0.22; *P* = 0.01) compared with the group with non-elevated levels at both times of assessment. The association persisted after adjustment for relevant confounders. Elevated ESR values at both times of assessment were not significantly associated with change in knee muscle strength (β = -0.05; *P* = 0.49).

**Conclusions:**

Our results indicate that elevated CRP values are related to a lower gain in muscle strength over time in patients with established knee OA. Although the mechanism to explain this relationship is not fully elucidated, these results suggest inflammation as a relevant factor influencing muscle strength in this group of patients.

## Introduction

In patients with knee osteoarthritis (OA), lower muscle strength is associated with disease progression and activity limitations [1-3]. Lower muscle strength in this group of patients is determined by several factors, including pain, avoidance of activities, and aging [4,5]. More recently, evidence from cross-sectional studies has suggested that inflammation is an additional factor influencing muscle strength in patients with knee OA [6,7].

A low grade of inflammation has been reported [8-10] in patients with OA, and slight or moderate elevations of inflammatory markers such as C-reactive protein (CRP) and erythrocyte sedimentation rate (ESR) have been described [8,11-15]. Recently, two cross-sectional studies have found an association between elevated inflammatory markers and lower muscle strength in patients with knee OA [6,7]. Although the mechanism has not been fully clarified, the association between elevated levels of inflammatory markers and muscle strength might be explained by the catabolic effect of inflammatory markers on muscle tissue [16]. Elevated levels of inflammatory markers might also contribute to reducing the muscle’s regenerative potential, probably through lowering protein synthesis rates [17]. Longitudinal studies analyzing the association of long-term inflammation with changes in muscle strength in patients with OA are needed in order to better understand the influence of inflammation on muscle strength. We hypothesized that if in patients with knee OA inflammatory markers (i.e., CRP and ESR) are elevated at both baseline and 2-year follow-up, this will be associated with a lower gain or decrease in muscle strength compared with patients who do not have elevated levels at both assessments.

## Methods

### Subjects

**Figure 1:**
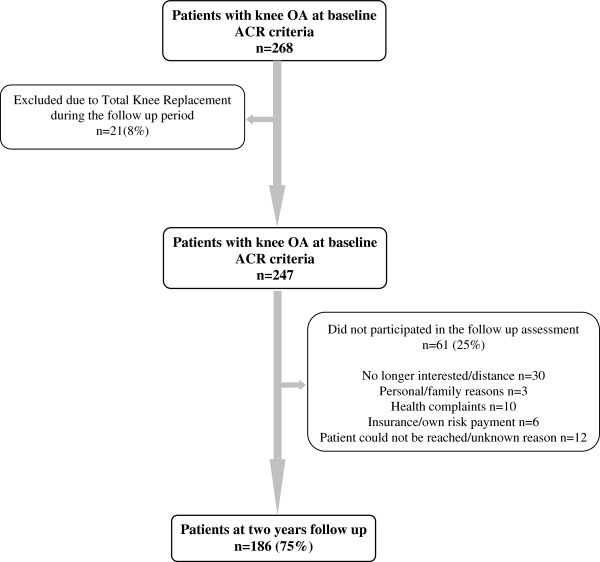
**Patient course during the study.** ACR, American College of Rheumatology; OA, osteoarthritis.

One hundred eighty-six participants from the Amsterdam Osteoarthritis (AMS-OA) cohort (127 females and 59 males) with unilateral or bilateral diagnosis of knee OA according to the American College of Rheumatology [18] were included in this study (2009 to 2011). The AMS-OA is a cohort of patients with OA of the knee or hip or both [18,19], who have been referred to an outpatient rehabilitation center (Reade, Centre for Rehabilitation and Rheumatology, Amsterdam, The Netherlands) [6]. Participants were assessed by rheumatologists, radiologists, and rehabilitation physicians. Rheumatoid arthritis (RA) or any other form of inflammatory arthritis (that is, crystal arthropathy, septic arthritis, and spondyloarthropathy) was considered an exclusion criterion. Total knee replacement during the follow-up period was considered an additional exclusion criterion. Demographic, clinical, radiographic, psychosocial, and biomechanical factors related to OA were assessed both at baseline and at 2-year follow-up. Patients from the AMS-OA cohort who had completed 2 years since the baseline measurements were invited to the follow-up assessment (2011 to 2013) (Figure 1). Only patients who completed the assessment at both times were included in the study. All of the participants provided written informed consent in accordance with the Declaration of Helsinki. The study was approved by the Reade Institutional Review Board.

### Measures

#### Inflammatory markers

Inflammatory markers were measured in serum from patients’ blood samples at baseline and at 2-year follow-up. CRP (in milligrams per liter) was processed immunoturbidimetrically by using CRPLX test kits [20,21] and the Roche Cobas-6000 analyzer (Roche Diagnostics GmbH, Mannheim, Germany). ESR values were determined by the standard Westergren method [22]. In this method, ethylenediaminetetraacetic acid (EDTA) anticoagulated blood samples were pre-diluted with saline solution and aspirated into the Westergren pipette graduated from 0 to 200 mm. The rate at which red blood cells sedimented in 1 hour was measured and reported in millimeters per hour.

#### Muscle strength

Knee muscle strength was assessed by using an isokinetic dynamometer (EnKnee; Enraf-Nonius, Rotterdam, The Netherlands) at baseline and at 2-year follow-up [23]. An initial practice attempt was used for the patients to get familiar with the required movements. The patients performed three maximal test repetitions to measure the isokinetic strength of the quadriceps and hamstrings for each knee at 60° per second. Mean quadriceps and hamstring muscle strength per leg was calculated (in Newton meters) and divided by the patient’s weight (in kilograms) [6,24]. This measure (in Newton meters per kilogram) has shown an excellent intra-rater reliability (intraclass correlation coefficient of 0.93) in patients with knee OA [25]. Average muscle strength of both legs was used if both knees had OA; otherwise, muscle strength of the knee with OA was incorporated in the analyses.

#### Potential confounders

Demographic data (i.e., age and gender) were recorded. Information related to comorbidities was collected with the Cumulative Illness Rating Scale [26]. This instrument gathers information related to 13 body systems, scoring from 0 (none) to 4 (extremely severe) according to the severity of the condition. The number of diseases on which the patients scored a severity of 2 or higher was calculated. Non-steroidal anti-inflammatory drug (NSAID) use was dichotomized (yes or no). Body mass index (BMI) was calculated as body mass in kilograms divided by height in square meters. Kellgren-Lawrence (KL) scores of the knee were used to assess the radiographic severity of the disease [27]. The frequency at which the subjects usually performed physical activity of at least 30 minutes during the week [28] was assessed only at 2-year follow-up. The potential confounder effect of changes in comorbidities score, NSAID use, BMI [29], and KL score over the follow-up period and the information available about physical activity were considered in the analyses.

The pain subscale of the Western Ontario and McMaster Universities Osteoarthritis (WOMAC) questionnaire was included as a potential confounder in the models. The WOMAC questionnaire has five items related to pain, two items related to stiffness, and 17 items related to activity limitations [30]. Each item can be scored from 0 to 4. Higher scores represent worse outcome. The WOMAC stiffness and activity limitation subscales and information related to physical therapy treatment were not included in the models but were used to further describe the study population (Table 1). A validated Dutch version of WOMAC was used in this study [31]. Additionally, at 2-year follow-up, the patients were asked whether they had received physiotherapy treatment during the past 2 years.

**Table 1:** Characteristics of the study population (n = 186)

	**Baseline**		**2-year follow-up**	
	**Number**		**Number**	
Age, years	186	61 (7.3)	-	-
Female, n (%)	186	127 (68)	-	-
Radiographic OA-KL score ≥2, n (%)	184	130 (70)	186	123 (66)
WOMAC pain score, 0-20	183	7.9 (3.8)	185	7.0 (4.3)ª
WOMAC stiffness score, 0-8	179	3.7 (1.7)	186	3.5 (2.0)ª
WOMAC physical function score, 0-68	183	28.6 (13.4)	186	27.0 (15.3)
Comorbidities count, CIRS ≥2	184	0.8 (1.0)	175	1.1 (1.0)ª
NSAIDs, yes, n (%)	185	30 (16)	186	38 (20)
Body mass index, kg/m^2^	185	29.3 (5.5)	186	29.3 (5.4)
Physical activity ≥30 minutes, times per week	-	No information	183	3.0 (1.3)
PT treatment during the past 2 years, n (%)	-	No information	186	149 (80)
Knee muscle strength^b^, Nm/kg	177	0.88 (0.4)	183	0.95 (0.4)ª
Inflammatory markers				
C-reactive protein, mg/L	183		184	
Median, IQR		2 (1-4)		2 (1-3)
Mean, SD		3.4 (5.4)		2.9 (3.1)
>3 mg/L, n (%)		50 (27)		45 (24)
ESR, mm/hour	183		186	
Median, IQR		9 (5-15)		7 (4-12)
Mean, SD		11.6 (8.9)		9.5 (8.4)ª
≥20 mm/hour, n (%)		34 (18)		25 (13)

Values are presented as mean ± standard deviation (SD), unless stated otherwise. ªSignificant difference between baseline and follow-up assessment (*P* <0.05). ^b^Mean quadriceps and hamstring muscles in osteoarthritis (OA) knees. CIRS, Cumulative Illness Rating Scale; ESR, erythrocyte sedimentation rate; IQR, interquartile range; KL, Kellgren-Lawrence; NSAID, non-steroidal anti-inflammatory drug; PT, physiotherapy; WOMAC, Western Ontario and McMaster Universities Osteoarthritis.

### Statistical analysis

Descriptive statistics were used to characterize the study population at baseline and at 2-year follow-up. Percentages were used for categorical variables, medians (interquartile ranges, or IQRs), and means (standard deviations, or SDs) for continuous variables. McNemar tests and paired *t* tests were used to analyze the differences in the distribution of the variables at baseline and at 2-year follow-up (Table 1).

We dichotomized the original CRP and ESR values [6] at baseline and at 2-year follow-up. CRP was codified as 0 if levels in serum were low to intermediate (≤3 mg/L) and as 1 if levels were elevated (>3 mg/L). This cutoff level was chosen according to previous studies in patients with OA [11,32]. ESR was codified following the classification criteria of low to normal rate 0 (<20 mm/hour) and elevated rate 1 (≥20 mm/hour) [19]. Based on the dichotomization previously described, overall inflammatory markers (i.e., CRP and ESR) were categorized in four subgroups as follows: (a) the values in serum were low to intermediate/normal at both times of measurement, (b) high values at baseline and decreased at 2-year follow-up, (c) low to intermediate/normal baseline values and increased at 2-year follow-up, and (d) the values were elevated at baseline and at follow-up. Change in muscle strength was calculated as the difference between follow-up and baseline muscle strength (in Newton meters per kilogram).

The association of inflammatory markers (CRP and ESR) as independent factors (subgroups described above) and change in muscle strength (in Newton meters per kilogram) as dependent factor was analyzed by using linear regression models. The group of patients with not elevated inflammatory markers at both baseline and follow-up was used as a reference. First, regression analyses were used to analyze the association of CRP and ESR with change in muscle strength adjusting for baseline muscle strength (crude models). Second, multivariable regression models adding one relevant confounding variable at a time (i.e., age, gender, change in comorbidities, change in NSAID use, change in BMI, change in WOMAC pain, and level of physical activity) were analyzed (Table 2). Statistical significance was accepted at *P* values of less than 0.05. All analyses were performed by using SPSS software, version 18.0 (SPSS Inc., Chicago, IL, USA).

**Table 2:** Associations of C-reactive protein with muscle strength change in patients with knee osteoarthritis

**Independent factors**	**Muscle strength change, N-m/kg**	
	**β**	***P*** **value**
CRP (crude)		
Not elevated at baseline and at follow-up	Ref.	
Elevated at baseline and not elevated at follow-up	0.05	0.51
Not elevated at baseline and elevated at follow-up	-0.09	0.22
Elevated at both times of assessment	-0.17	0.02
CRP^a,b^		
Not elevated at baseline and at follow-up	Ref.	
Elevated at baseline and not elevated at follow-up	0.06	0.38
Not elevated at baseline and elevated at follow-up	-0.06	0.38
Elevated at both times of assessment	-0.17	0.02
CRP^a-c^		
Not elevated at baseline and at follow-up	Ref.	
Elevated at baseline and not elevated at follow-up	0.06	0.44
Not elevated at baseline and elevated at follow-up	-0.06	0.42
Elevated at both times of assessment	-0.16	0.03
CRP^a-d^		
Not elevated at baseline and at follow-up	Ref.	
Elevated at baseline and not elevated at follow-up	0.03	0.71
Not elevated at baseline and elevated at follow-up	-0.07	0.38
Elevated at both times of assessment	-0.18	0.02
CRP^a-e^		
Not elevated at baseline and at follow-up	Ref.	
Elevated at baseline and not elevated at follow-up	0.01	0.92
Not elevated at baseline and elevated at follow-up	-0.09	0.25
Elevated at both times of assessment	-0.20	0.01
CRP^a-f^		
Not elevated at baseline and at follow-up	Ref.	
Elevated at baseline and not elevated at follow-up	0.01	0.90
Not elevated at baseline and elevated at follow-up	-0.09	0.26
Elevated at both times of assessment	-0.19	0.01
CRP^a-g^		
Not elevated at baseline and at follow-up	Ref.	
Elevated at baseline and not elevated at follow-up	0.01	0.93
Not elevated at baseline and elevated at follow-up	-0.09	0.25
Elevated at both times of assessment	-0.19	0.01
CRP^a-h^		
Not elevated at baseline and at follow-up	Ref.	
Elevated at baseline and not elevated at follow-up	0.05	0.57
Not elevated at baseline and elevated at follow-up	-0.09	0.25
Elevated at both times of assessment	-0.21	0.01

This linear regression analysis used muscle strength change as a dependent factor. All of the models were adjusted for baseline muscle strength. Additionally, confounders adjusting the model were ^a^age, ^b^gender, ^c^comorbidities change, ^d^non-steroidal anti-inflammatory drug change, ^e^body mass index change, ^f^Western Ontario and McMaster Universities Osteoarthritis pain change, ^g^Kellgren-Lawrence score change, and ^h^physical activity follow-up. Reduce patient numbers in the multivariate linear regression analyses due to random missing in the outcome measure or selected predictors. CRP, C-reactive protein; Ref., reference.

## Results

### Participants

In total, 268 patients with knee OA who completed the baseline were invited to participate in the follow-up evaluation. Eight percent of the patients (n = 21) were excluded from the study because of total knee replacement. From the eligible subjects who met the inclusion criteria at follow-up (n = 247), 25% (n = 61) declined the invitation for various reasons. Figure 1 shows the participant flow during the study. There were non-significant differences in baseline characteristics between the groups of patients who were and those who were not part of the 2-year follow-up assessment (data no shown).

### Descriptives

Demographic and clinical characteristic data of patients who participated at baseline and at 2-year follow-up (n = 186) are shown in Table 1. Sixty-eight percent of the study group (n = 127) were women. Mean ± SD age at baseline was 61.2 ± 7.3 years. The median (IQR) values of CRP and ESR in the group were 2 mg/L (1 to 4) and 9 mm/hour (5 to 15) at baseline and 2 mg/L (1 to 3) and 7 mm/hour (4 to 12) at 2-year follow-up, respectively. Sixty-four percent of the study population (n = 117) had not elevated CRP at both baseline and 2-year follow-up, 11% (n = 20) had elevated CRP values at baseline and not elevated at follow-up, 9% (n = 16) had not elevated CRP values at baseline and elevated levels at follow-up, and 16% (n = 29) of the study population had elevated CRP levels at both times of measurement. Seventy-five percent (n = 140) of the patients had not elevated ESR values at both baseline and follow-up, 10% (n = 18) had elevated ESR values at baseline and not elevated at follow-up, 5% (n = 9) had not elevated ESR values at baseline and elevated levels at follow-up, and 9% (n = 16) of the study group had elevated levels of ESR at both times of assessment. Data on CRP and ESR were missing at one point of assessment in four and three patients, respectively.

There was an overall 19% increase in mean knee muscle strength in the study group at follow-up (mean ± SD of 0.09 ± 0.2 N-m/kg) (*P* <0.001). Patients without elevated levels of CRP at both baseline and follow-up had a mean muscle strength increase of 27% at 2-year follow-up (mean ± SD of 0.10 ± 0.3 N-m/kg) (*P* <0.001); patients with elevated CRP levels at both times of assessment showed a non-significant increase in mean muscle strength of only 4% (mean ± SD of 0.02 ± 0.2 N-m/kg) (*P* = 0.59). In patients with only one knee affected with OA, there was a non-significant difference in knee muscle strength change at 2 years between the knee with OA and the one without the disease (*P* = 0.19) (data not shown).

### Associations of inflammatory markers (C-reactive protein and erythrocyte sedimentation rate) with muscle strength changes

Tables 2 and 3 show the associations of the CRP and ESR (subgroups) with muscle strength change using the group of not elevated inflammatory markers at both baseline and follow-up as a reference. Elevated CRP values at both baseline and 2-year follow-up (crude β -0.17; *P* = 0.02), but not ESR (crude β -0.11; *P* = 0.16), were significantly associated with a lower increase in muscle strength after 2 years compared with the reference group. In the multivariable regression models, one relevant confounding variable was added at a time (Tables 2 and 3). In the fully adjusted model, elevated levels of CRP at both times of assessment (β -0.21; *P* = 0.01), but not ESR (β -0.07; *P* = 0.38), were still associated with lower gains in muscle strength, compared with the reference group, after adjustment for baseline muscle strength, age, gender, change in comorbidities, change in NSAID use, change in BMI, change in WOMAC pain, KL score change, and level of physical activity. The coefficients of the full model were not affected once the number of knees affected with OA was incorporated in the full model (data not shown). There were no statistically significant differences in muscle strength changes in the groups with inflammatory markers (CRP or ESR) elevated at only one point of assessment (baseline or follow-up) compared with the reference group, before or after adjustment for relevant confounders.

**Table 3:** Associations of erythrocyte sedimentation rate with muscle strength change in patients with knee osteoarthritis

**Independent factors**	**Muscle strength change, N-m/kg**	
	**β**	***P*** **value**
ESR (crude)		
Not elevated at baseline and at follow-up	Ref.	
Elevated at baseline and not elevated at follow-up	-0.07	0.34
Not elevated at baseline and elevated at follow-up	-0.05	0.48
Elevated at both times of assessment	-0.11	0.16
ESR^a,b^		
Not elevated at baseline and at follow-up	Ref.	
Elevated at baseline and not elevated at follow-up	-0.06	0.39
Not elevated at baseline and elevated at follow-up	-0.05	0.46
Elevated at both times of assessment	-0.09	0.19
ESR^a-c^		
Not elevated at baseline and at follow-up	Ref.	
Elevated at baseline and not elevated at follow-up	-0.06	0.41
Not elevated at baseline and elevated at follow-up	-0.01	0.95
Elevated at both times of assessment	-0.08	0.26
ESR^a-d^		
Not elevated at baseline and at follow-up	Ref.	
Elevated at baseline and not elevated at follow-up	-0.08	0.28
Not elevated at baseline and elevated at follow-up	0.01	0.99
Elevated at both times of assessment	-0.08	0.27
ESR^a-e^		
Not elevated at baseline and at follow-up	Ref.	
Elevated at baseline and not elevated at follow-up	-0.09	0.24
Not elevated at baseline and elevated at follow-up	0.04	0.96
Elevated at both times of assessment	-0.08	0.31
ESR^a-f^		
Not elevated at baseline and at follow-up	Ref.	
Elevated at baseline and not elevated at follow-up	-0.10	0.20
Not elevated at baseline and elevated at follow-up	-0.03	0.76
Elevated at both times of assessment	-0.07	0.35
ESR^a-g^		
Not elevated at baseline and at follow-up	Ref.	
Elevated at baseline and not elevated at follow-up	-0.10	0.19
Not elevated at baseline and elevated at follow-up	-0.01	0.86
Elevated at both times of assessment	-0.07	0.34
ESR^a-h^		
Not elevated at baseline and at follow-up	Ref.	
Elevated at baseline and not elevated at follow-up	-0.10	0.17
Not elevated at baseline and elevated at follow-up	-0.02	0.79
Elevated at both times of assessment	-0.07	0.38

This linear regression analysis used muscle strength change as a dependent factor. All the models were adjusted for baseline muscle strength. Additionally, confounders adjusting the model were ^a^age, ^b^gender, ^c^comorbidities change, ^d^non-steroidal anti-inflammatory drug change, ^e^body mass index change, ^f^Western Ontario and McMaster Universities Osteoarthritis pain change, ^g^Kellgren-Lawrence score change, and ^h^physical activity follow-up. Reduce patients numbers in the multivariate linear regression analyses due to random missing in the outcome measure or selected predictors. ESR, erythrocyte sedimentation rate; Ref., reference.

## Discussion

This study investigated the association of elevated serum inflammatory markers (i.e., CRP and ESR) and changes in knee muscle strength in a group of patients with established knee OA, for a period of 2 years. We found that a persistently elevated level of serum CRP values was associated with lower gain in muscle strength compared with patients with not elevated levels at both times of assessment. Previous studies have documented the cross-sectional relationship between elevated inflammatory markers and lower muscle strength in patients with knee OA [6,7]. However, to the best of our knowledge, this is the first study on the longitudinal association between levels of serum inflammatory and knee muscle strength in patients with knee OA.

There was an overall increase in knee muscle strength after 2 years in the study population. The improvement of muscle strength could be explained by the fact that the patients in this study were initially referred to our outpatient rehabilitation center to receive medical attention, and 80% of the study population reported that they had received some type of physical therapy intervention during the follow-up period. This may have resulted in increased muscle strength.

The relationship between elevated inflammatory markers and lower knee muscle strength is consistent with findings from previous cross-sectional studies carried out in patients with OA [6,7,33], in patients with RA [34], and in the general older population. Schaap and colleagues [35] described an association between baseline elevated serum inflammatory markers and a decline in knee and hand strength after 5 years in a group of older adults [36].

In the present study, when change in BMI [29] at 2 years was incorporated in the fully adjusted model, elevated levels of serum CRP at both baseline and 2-year follow-up were still significantly associated with lower gain in muscle strength compared with patients with not elevated levels at both times of assessment. Nevertheless, in our previous cross-sectional study, the association between inflammatory markers and muscle strength was not independent of BMI [6]. A possible production and secretion of several pro-inflammatory cytokines by the adipose tissue or a non-hepatic production of CRP through the stimulation of adiposities or both might help to explain the strong association, previously reported, between systemic inflammatory markers and BMI [32,37,38]. BMI is a relevant confounder in the association between inflammatory markers and muscle strength. However, small changes in BMI over time do not influence the association between elevated inflammatory markers and change in muscle strength.

In older adults, elevated levels of inflammatory markers have been associated not only with lower muscle strength but also with loss of muscle mass and sarcopenia [29,35,39]. These associations might be explained by the catabolic effect of inflammatory markers on muscle tissue [16,40]. Higher levels of inflammatory factors might contribute to reducing the muscle’s regenerative potential, probably through lowering protein synthesis rates in the skeletal muscle [17]. Additionally, results from studies carried out in rats suggested that inflammatory factors might cause muscle breakdown [41,42]. Bodell and colleagues [43] indicated that long exposure of skeletal muscle to interleukin-6 (IL-6) can retard muscle growth in rats, possibly due to the interaction with key growth factors. Although these mechanisms have not been intensively studied in humans, the same mechanism found in rat models might be involved in the development of muscle weakness in patients with knee OA.

The changes in muscle strength after 2 years were not associated with persistently elevated levels of serum ESR. A possible explanation is that ESR can be affected by multiple factors such as gender, age, temperature, smoking, levels of plasma proteins, and red blood cell factors [44]. Additionally, ESR is less sensitive than CRP to changes in the onset of acute inflammation and has shown low/moderate reproducibility with a wide range of normal results compared with the high reproducibility of CRP with a narrow range of normal results. Looking at different inflammatory markers—i.e., IL-6 and tumor necrosis factor (TNF)—in future studies might provide further support for the long-term association between inflammation and muscle strength.

There were no statistically significant differences in muscle strength changes in the groups with elevated inflammatory markers at only one point of assessment (baseline or follow-up) compared with the reference group (not elevated levels at both baseline and follow-up), before or after adjusting for relevant confounders. We suggest that longer exposure of skeletal muscle tissue to elevated levels of inflammatory markers might be required to induce changes in muscle strength, especially when using systemic inflammatory markers such as CRP, which is highly sensitive to changes in the onset of acute-phase response. On the other hand, it is important to consider that the small number of subjects in the groups with elevated inflammatory markers at only one time of assessment might have affected these results.

Some limitations of this study have to be considered. First, 25% of patients dropped out of the study at follow-up. However, the relevant baseline characteristics were not statistically different between patients who completed and those who did not complete the follow-up assessment, and this makes us believe that this loss of patients at follow-up did not impact the results of our study. Second, although this is a longitudinal study, we can prove only that associations exist, but it is not possible to establish the causality underlying them. Therefore, further experimental studies should be undertaken to clarify the causality. Third, the absence of standard conditions at the time of the blood collection (i.e., fasting conditions, preceding physical activity, time of sample collection, and so on) might have caused random variations in the levels of inflammatory markers. However, in the present study, blood samples were collected as part of the medical care, and additional parameters could not be controlled. Fourth, we gathered information on inflammatory markers and muscle strength at baseline and at 2-year follow-up, but we have no information about those variables in between these measurement points. Studies that include more than two time measurements might be necessary to further understand the longitudinal association between inflammation and muscle strength. Fifth, information about the percentage of body fat of the patients was not available. Therefore, we cannot conclude whether changes in fat mass during the follow-up period were or were not associated with changes in CRP and ESR levels. However, BMI, a proxy measure of body composition, was used as a potential cofounder in the present study. Sixth, in the present study, only knee muscle strength was assessed. However, other skeletal muscles might be affected by persistently elevated levels of inflammatory markers. A key strength of our study is the large number of patients with knee OA (n = 186) studied and the longitudinal design, compared with the smaller sample populations included in previous cross-sectional studies [7,45].

From a clinical perspective, the results of this study may contribute to more targeted treatment. Although further evidence is needed, targeting low-grade inflammation by pharmacological, nutritional, or lifestyle factors [46] or a combination of these might contribute to limiting sarcopenia and decreased muscle strength in patients with knee OA. On the other hand, it is possible that an increase in muscle strength (through muscle training) contributes to a decrease in circulating inflammatory markers in patients with OA. In this respect, previous literature has suggested that physical activity might contribute to mitigating inflammation [47]. Furthermore, decreases in circulating levels of inflammatory markers (i.e., CRP and TNF) were associated with an increase in muscle strength in a group of post-menopausal women receiving resistance training [48] as well as in older individuals with knee OA receiving a whole-body vibration program [49]. It is also feasible that both mechanisms are involved. Indeed, further longitudinal studies involving controlled and tailored interventions are needed to elucidate the clinical relevance of the association between inflammation and lower muscle strength in patients with knee OA.

## Conclusions

Our results indicate that persistently elevated CRP levels are independently related to a lower gain in muscle strength over time in patients with established knee OA. Although the mechanism to explain this relationship is not fully elucidated, these results suggest inflammation as a relevant factor influencing muscle strength in this group of patients.

## Abbreviations

AMS-OA: Amsterdam Osteoarthritis; BMI: body mass index; CRP: C-reactive protein; ESR: erythrocyte sedimentation rate; IL-6: interleukin-6; IQR: interquartile range; KL: Kellgren-Lawrence; NSAID: non-steroidal anti-inflammatory drug; OA: osteoarthritis; RA: rheumatoid arthritis; SD: standard deviation; TNF: tumor necrosis factor; WOMAC: Western Ontario and McMaster Universities Osteoarthritis.

## Competing interests

The authors declare that they have no competing interests.

## Authors’ contributions

DCS-R, MvdL, and JD participated in the conception and design of the study, contributed to the statistical analysis of data and to the interpretation and preparation of results, and helped to draft the manuscript. MvdE and WFL participated in the conception and design of the study. SV participated in the conception and design of the study and contributed to the statistical analysis of data and to the interpretation and preparation of results. LDR participated in the conception and design of the study and contributed to the acquisition of data, to the statistical analysis of data, and to the interpretation and preparation of results. JHvD contributed to the statistical analysis of data and to the interpretation and preparation of results. All authors read and approved the final manuscript.
